# *catena*-Poly[[lead(II)-bis­(μ_2_-quinolin-8-ol­ato)-κ^3^ *N*,*O*:*O*;κ^3^ *O*:*N*,*O*] *N*,*N*-di­methyl­formamide hemisolvate]

**DOI:** 10.1107/S1600536812002796

**Published:** 2012-01-31

**Authors:** Akbar Ghaemi, Zohreh Dadkhah, Seik Weng Ng, Edward R. T. Tiekink

**Affiliations:** a Department of Chemistry, Saveh Branch, Islamic Azad University, Saveh, Iran; b Department of Chemistry, University of Malaya, 50603 Kuala Lumpur, Malaysia, and Chemistry Department, Faculty of Science, King Abdulaziz University, PO Box 80203, Jeddah, Saudi Arabia; c Department of Chemistry, University of Malaya, 50603 Kuala Lumpur, Malaysia

## Abstract

The asymmetric unit of the title compound, {[Pb(C_9_H_6_NO)_2_]·0.5C_3_H_7_NO}_*n*_, comprises Pb(quinolate)_2_ and half a dimethyl­formamide mol­ecule (which is disordered about a centre of inversion). The quinolate ligands *N*,*O*-chelate to a Pb^II^ ion and simultaneously bridge a neighbouring Pb^II^ ion to form a polymeric chain along [100] comprising Pb-linked Pb_2_O_2_ distorted rhombi. These chains pack to form a square grid, with the channels thus defined occupied by the disordered solvent mol­ecules.

## Related literature

For a recent Pb^II^ mixed quinolate carboxyl­ate structure, see: Ghaemi *et al.* (2012 ▶). For the structure of the solvent-free Pb^II^ quinolate, see: Zhu *et al.* (2005 ▶).
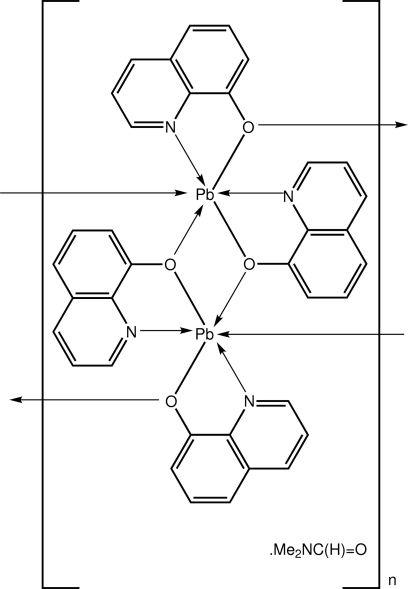


## Experimental

### 

#### Crystal data

[Pb(C_9_H_6_NO)_2_]·0.5C_3_H_7_NO
*M* *_r_* = 532.04
Triclinic, 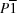

*a* = 8.1841 (2) Å
*b* = 9.6606 (3) Å
*c* = 10.8619 (3) Å
α = 96.683 (3)°
β = 98.277 (2)°
γ = 94.225 (3)°
*V* = 840.48 (4) Å^3^
*Z* = 2
Mo *K*α radiation
μ = 10.06 mm^−1^
*T* = 100 K
0.30 × 0.08 × 0.04 mm

#### Data collection

Agilent SuperNova Dual diffractometer with Atlas detector
Absorption correction: multi-scan (*CrysAlis PRO*; Agilent, 2010 ▶) *T*_min_ = 0.152, *T*_max_ = 0.689
13299 measured reflections
3866 independent reflections
3613 reflections with *I* > 2σ(*I*)
*R*_int_ = 0.036

#### Refinement

*R*[*F* ^2^ > 2σ(*F* ^2^)] = 0.021
*wR*(*F* ^2^) = 0.049
*S* = 1.01
3866 reflections
272 parameters
36 restraints
H-atom parameters constrained
Δρ_max_ = 1.23 e Å^−3^
Δρ_min_ = −0.71 e Å^−3^

### 

Data collection: *CrysAlis PRO* (Agilent, 2010 ▶); cell refinement: *CrysAlis PRO*; data reduction: *CrysAlis PRO*; program(s) used to solve structure: *SHELXS97* (Sheldrick, 2008 ▶); program(s) used to refine structure: *SHELXL97* (Sheldrick, 2008 ▶); molecular graphics: *ORTEP-3* (Farrugia, 1997 ▶) and *DIAMOND* (Brandenburg, 2006 ▶); software used to prepare material for publication: *publCIF* (Westrip, 2010 ▶).

## Supplementary Material

Crystal structure: contains datablock(s) global, I. DOI: 10.1107/S1600536812002796/hg5166sup1.cif

Structure factors: contains datablock(s) I. DOI: 10.1107/S1600536812002796/hg5166Isup2.hkl

Additional supplementary materials:  crystallographic information; 3D view; checkCIF report

## Figures and Tables

**Table 1:** Selected bond lengths (Å)

Pb—O2	2.408 (2)
Pb—O1	2.468 (2)
Pb—N2	2.470 (3)
Pb—N1	2.566 (3)
Pb—O1^i^	2.618 (2)
Pb—O2^ii^	2.812 (2)

Symmetry codes: (i) 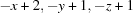; (ii) 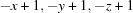.

**Fig. 1.**
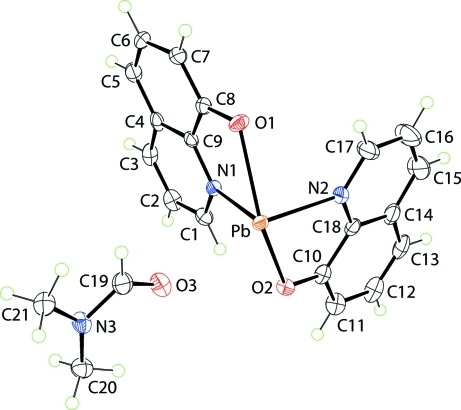
The asymmetric unit of (I) showing the atom-labelling scheme and displacement ellipsoids at the 50% probability level.

**Fig. 2.**
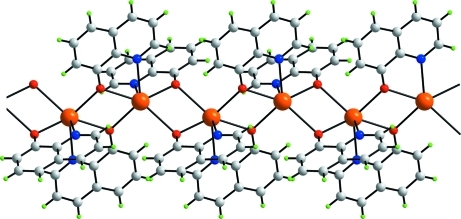
A view of the polymeric chain along [100] in (I).

**Fig. 3.**
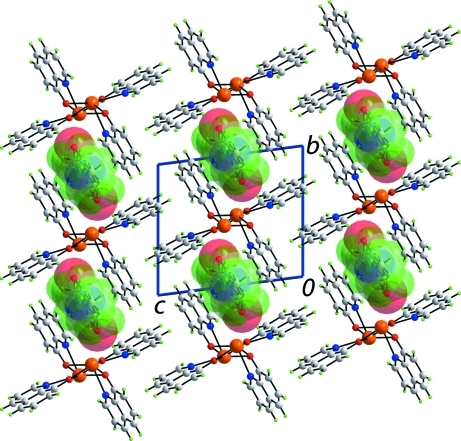
A view in projection down the a axis of the unit-cell contents of (I) highlighting the square grid defined by the polymeric chains and the inclusion of the solvent molecules (shown in space-filling mode) in the channels.

## supplementary crystallographic information

### Comment

During the course of recent studies into the structural chemistry of mixed Pb^II^ quinolate carboxylates (Ghaemi *et al.*, 2012), the title binary Pb^II^ quinolate was isolated as a DMF hemi-solvate, (I), from an attempted reaction with maleic acid. The crystal structure of the solvent free and polymeric Pb(quinolate)_2_ has been described (Zhu *et al.*, 2005).

The asymmetric unit of (I) comprises Pb(quinolate)_2_ and half a solvent DMF molecule (this is disordered over a centre of inversion), Fig. 1. Each quinolate anion *N*,*O*-chelates a Pb^II^ atom and at the same time bridges a neighbouring Pb^II^ atom *via* the carbonyl-O atom. The result is a polymeric chain comprising alternating Pb_2_O_2_ rhombi, Fig. 2. The degree in asymmetry in the Pb—O bridges varies, Table 1. The coordination geometry of the Pb^II^ atom is based on a distorted pentagonal bipyramid with one N atom occupying an axial site. The lone pair of electrons occupies the second axial position. It is noted that the O3 and O3' atoms (each with a 0.25 site occupancy factor) of disordered DMF molecule approach the Pb^II^ at distances 2.903 (12) and 2.977 (12) Å, respectively. These are not *trans* to the axial N atom forming angles of approximately 140°. If one of the DMF-O atoms is included as part of the coordination sphere, the coordination geometry would be described as ψ-dodecahedral.

In the crystal packing, the polymeric chains pack into a square grid which defines channels in which reside the disordered solvent molecules, Fig. 3. The aforementioned weak Pb···O(DMF) interactions serve to connect the polymeric chains into a layer in the *ab* plane.

### Experimental

The title complex was obtained by the following method. 8-Hydroxyquinoline (0.036 g, 0.25 mmol) was added to an aqueous solution (5 ml) of Pb(NO_3_)_2_ (0.082 g, 0.25 mmol). The mixture was stirred for 15 min. Then to this solution, a DMF solution (5 ml) of maleic acid (0.029 g, 0.25 mmol) which with triethylamine neutralized was added slowly at room temperature. This mixture was filtered. After keeping the filtrate in air, crystals were formed at the bottom of the vessel on slow evaporation of the solvents at room temperature. M.p. 590 K. Yield: 65%.

### Refinement

Carbon-bound H atoms were placed in calculated positions [C—H 0.95–0.98 Å, *U*_iso_(H) 1.2–1.5*U*_eq_(C)] and were included in the refinement in the riding model approximation.

The DMF molecule is disordered over two sites over a centre of inversion. The C—O distances were restrained to 1.25 (1) Å, the C_carbonyl_—N distances to 1.35 (1) Å and the C_methyl_—N distances to 1.45 (1) Å. Each component was restrained to lie on a plane; the anisotropic displacement parameters of the primed atoms were set to those of the unprimed ones, and they were tightly restrained to be nearly isotropic.

The final difference Fourier map had a peak of 1.23 Å^-3^ at 1.10 Å from the Pb atom.

### Crystal data

**Table:**

[Pb(C_9_H_6_NO)_2_]·0.5C_3_H_7_NO	*Z* = 2
*M**_r_* = 532.04	*F*(000) = 504
Triclinic, *P*1	*D*_x_ = 2.102 Mg m^−^^3^
Hall symbol: -P 1	Mo *K*α radiation, λ = 0.71073 Å
*a* = 8.1841 (2) Å	Cell parameters from 8798 reflections
*b* = 9.6606 (3) Å	θ = 2.5–27.5°
*c* = 10.8619 (3) Å	µ = 10.06 mm^−^^1^
α = 96.683 (3)°	*T* = 100 K
β = 98.277 (2)°	Block, yellow
γ = 94.225 (3)°	0.30 × 0.08 × 0.04 mm
*V* = 840.48 (4) Å^3^	

### Data collection

**Table:**

Agilent SuperNova Dual diffractometer with Atlas detector	3866 independent reflections
Radiation source: SuperNova (Mo) X-ray Source	3613 reflections with *I* > 2σ(*I*)
Mirror	*R*_int_ = 0.036
Detector resolution: 10.4041 pixels mm^-1^	θ_max_ = 27.6°, θ_min_ = 2.5°
ω scans	*h* = −10→10
Absorption correction: multi-scan (CrysAlis PRO; Agilent, 2010)	*k* = −12→12
*T*_min_ = 0.152, *T*_max_ = 0.689	*l* = −14→14
13299 measured reflections	

### Refinement

**Table:**

Refinement on *F*^2^	Primary atom site location: structure-invariant direct methods
Least-squares matrix: full	Secondary atom site location: difference Fourier map
*R*[*F*^2^ > 2σ(*F*^2^)] = 0.021	Hydrogen site location: inferred from neighbouring sites
*wR*(*F*^2^) = 0.049	H-atom parameters constrained
*S* = 1.01	*w* = 1/[σ^2^(*F*_o_^2^) + (0.0245*P*)^2^ + 0.0115*P*] where *P* = (*F*_o_^2^ + 2*F*_c_^2^)/3
3866 reflections	(Δ/σ)_max_ = 0.002
272 parameters	Δρ_max_ = 1.23 e Å^−^^3^
36 restraints	Δρ_min_ = −0.71 e Å^−^^3^

### Special details

**Table:**

Geometry. All e.s.d.'s (except the e.s.d. in the dihedral angle between two l.s. planes) are estimated using the full covariance matrix. The cell e.s.d.'s are taken into account individually in the estimation of e.s.d.'s in distances, angles and torsion angles; correlations between e.s.d.'s in cell parameters are only used when they are defined by crystal symmetry. An approximate (isotropic) treatment of cell e.s.d.'s is used for estimating e.s.d.'s involving l.s. planes.
Refinement. Refinement of *F*^2^ against ALL reflections. The weighted *R*-factor *wR* and goodness of fit *S* are based on *F*^2^, conventional *R*-factors *R* are based on *F*, with *F* set to zero for negative *F*^2^. The threshold expression of *F*^2^ > σ(*F*^2^) is used only for calculating *R*-factors(gt) *etc*. and is not relevant to the choice of reflections for refinement. *R*-factors based on *F*^2^ are statistically about twice as large as those based on *F*, and *R*-factors based on ALL data will be even larger.

### Fractional atomic coordinates and isotropic or equivalent isotropic displacement parameters (Å^2^)

**Table:**

	*x*	*y*	*z*	*U*_iso_*/*U*_eq_	Occ. (<1)
Pb	0.758370 (14)	0.465630 (13)	0.543410 (11)	0.01554 (5)	
O1	1.0552 (3)	0.4449 (3)	0.6130 (2)	0.0232 (6)	
O2	0.5307 (3)	0.5563 (3)	0.6350 (2)	0.0180 (5)	
N1	0.8296 (3)	0.4221 (3)	0.7727 (3)	0.0162 (6)	
N2	0.8215 (4)	0.7122 (3)	0.6385 (3)	0.0249 (7)	
C1	0.7215 (4)	0.4163 (4)	0.8528 (3)	0.0206 (8)	
H1	0.6154	0.4481	0.8308	0.025*	
C2	0.7575 (5)	0.3651 (4)	0.9686 (3)	0.0248 (8)	
H2	0.6775	0.3630	1.0238	0.030*	
C3	0.9089 (5)	0.3184 (4)	1.0005 (3)	0.0247 (8)	
H3	0.9344	0.2833	1.0784	0.030*	
C4	1.0284 (4)	0.3217 (4)	0.9190 (3)	0.0204 (8)	
C5	1.1862 (5)	0.2725 (4)	0.9433 (4)	0.0259 (8)	
H5	1.2177	0.2343	1.0190	0.031*	
C6	1.2937 (5)	0.2792 (4)	0.8591 (4)	0.0296 (9)	
H6	1.3985	0.2434	0.8756	0.036*	
C7	1.2512 (4)	0.3389 (4)	0.7477 (4)	0.0253 (8)	
H7	1.3301	0.3448	0.6920	0.030*	
C8	1.0982 (4)	0.3891 (4)	0.7165 (3)	0.0193 (7)	
C9	0.9830 (4)	0.3776 (4)	0.8040 (3)	0.0178 (7)	
C10	0.9624 (5)	0.7881 (5)	0.6391 (6)	0.0462 (14)	
H10	1.0427	0.7492	0.5938	0.055*	
C11	0.9986 (6)	0.9237 (5)	0.7038 (7)	0.071 (2)	
H11	1.1016	0.9753	0.7023	0.086*	
C12	0.8836 (6)	0.9807 (5)	0.7689 (6)	0.0586 (17)	
H12	0.9070	1.0722	0.8134	0.070*	
C13	0.7312 (5)	0.9046 (4)	0.7703 (4)	0.0306 (9)	
C14	0.6057 (5)	0.9570 (4)	0.8344 (4)	0.0311 (9)	
H14	0.6214	1.0486	0.8795	0.037*	
C15	0.4614 (5)	0.8749 (4)	0.8309 (4)	0.0299 (9)	
H15	0.3775	0.9103	0.8744	0.036*	
C16	0.4342 (5)	0.7391 (4)	0.7644 (4)	0.0263 (9)	
H16	0.3319	0.6856	0.7640	0.032*	
C17	0.5523 (4)	0.6806 (4)	0.6993 (3)	0.0188 (7)	
C18	0.7048 (4)	0.7676 (4)	0.7030 (3)	0.0199 (7)	
O3	0.5643 (14)	0.2137 (13)	0.5793 (11)	0.0316 (18)	0.25
N3	0.476 (2)	−0.011 (2)	0.4938 (14)	0.0236 (17)	0.25
C19	0.588 (2)	0.1025 (17)	0.5172 (12)	0.031 (4)	0.25
H19	0.6900	0.0971	0.4850	0.037*	0.25
C20	0.319 (3)	−0.009 (3)	0.540 (2)	0.026 (4)	0.25
H20A	0.3237	−0.0606	0.6134	0.039*	0.25
H20B	0.2305	−0.0539	0.4745	0.039*	0.25
H20C	0.2977	0.0877	0.5650	0.039*	0.25
C21	0.557 (2)	−0.112 (2)	0.4205 (18)	0.026 (4)	0.25
H21A	0.4848	−0.1461	0.3412	0.039*	0.25
H21B	0.5803	−0.1910	0.4675	0.039*	0.25
H21C	0.6618	−0.0679	0.4030	0.039*	0.25
O3'	0.7253 (14)	0.1552 (12)	0.5310 (11)	0.0316 (18)	0.25
N3'	0.492 (3)	0.0002 (19)	0.4949 (14)	0.0236 (17)	0.25
C19'	0.5783 (18)	0.124 (2)	0.5450 (16)	0.031 (4)	0.25
H19'	0.5247	0.1905	0.5929	0.037*	0.25
C20'	0.511 (3)	−0.130 (2)	0.4189 (19)	0.026 (4)	0.25
H20D	0.4190	−0.1498	0.3493	0.039*	0.25
H20E	0.5115	−0.2068	0.4708	0.039*	0.25
H20F	0.6162	−0.1220	0.3856	0.039*	0.25
C21'	0.323 (3)	−0.023 (3)	0.518 (2)	0.026 (4)	0.25
H21D	0.3170	−0.0921	0.5772	0.039*	0.25
H21E	0.2505	−0.0590	0.4388	0.039*	0.25
H21F	0.2859	0.0649	0.5533	0.039*	0.25

### Atomic displacement parameters (Å^2^)

**Table:**

	*U*^11^	*U*^22^	*U*^33^	*U*^12^	*U*^13^	*U*^23^
Pb	0.01436 (7)	0.01590 (8)	0.01590 (8)	0.00224 (5)	0.00048 (5)	0.00189 (5)
O1	0.0172 (12)	0.0320 (15)	0.0241 (14)	0.0070 (11)	0.0044 (10)	0.0146 (12)
O2	0.0180 (12)	0.0203 (13)	0.0154 (13)	0.0008 (10)	0.0042 (10)	−0.0007 (10)
N1	0.0178 (14)	0.0164 (15)	0.0138 (15)	0.0004 (11)	0.0001 (11)	0.0032 (12)
N2	0.0189 (16)	0.0168 (16)	0.040 (2)	0.0027 (12)	0.0041 (14)	0.0068 (15)
C1	0.0215 (18)	0.0200 (19)	0.0199 (19)	−0.0006 (14)	0.0047 (14)	−0.0002 (15)
C2	0.034 (2)	0.024 (2)	0.0167 (19)	−0.0017 (16)	0.0065 (16)	0.0016 (16)
C3	0.039 (2)	0.0175 (19)	0.0156 (18)	0.0003 (16)	−0.0020 (16)	0.0022 (15)
C4	0.0273 (19)	0.0124 (17)	0.0176 (18)	−0.0047 (14)	−0.0054 (14)	0.0012 (14)
C5	0.028 (2)	0.021 (2)	0.025 (2)	−0.0033 (15)	−0.0080 (16)	0.0071 (16)
C6	0.0201 (19)	0.022 (2)	0.047 (3)	0.0030 (15)	−0.0066 (17)	0.0151 (19)
C7	0.0186 (18)	0.025 (2)	0.034 (2)	0.0006 (15)	0.0031 (16)	0.0136 (18)
C8	0.0185 (17)	0.0180 (18)	0.0213 (19)	−0.0001 (14)	−0.0001 (14)	0.0072 (15)
C9	0.0184 (17)	0.0130 (17)	0.0195 (18)	−0.0032 (13)	−0.0028 (13)	0.0019 (14)
C10	0.021 (2)	0.024 (2)	0.095 (4)	0.0009 (17)	0.013 (2)	0.005 (3)
C11	0.031 (3)	0.021 (2)	0.158 (7)	−0.003 (2)	0.015 (3)	−0.003 (3)
C12	0.041 (3)	0.014 (2)	0.113 (5)	−0.0008 (19)	−0.002 (3)	−0.007 (3)
C13	0.035 (2)	0.0173 (19)	0.036 (2)	0.0053 (16)	−0.0092 (18)	0.0034 (18)
C14	0.048 (3)	0.019 (2)	0.024 (2)	0.0141 (18)	−0.0063 (18)	−0.0012 (17)
C15	0.049 (3)	0.031 (2)	0.0136 (19)	0.0164 (19)	0.0081 (17)	0.0035 (17)
C16	0.034 (2)	0.029 (2)	0.0175 (19)	0.0051 (17)	0.0097 (16)	0.0032 (17)
C17	0.0270 (19)	0.0208 (18)	0.0089 (16)	0.0056 (15)	0.0017 (14)	0.0023 (14)
C18	0.0213 (18)	0.0189 (18)	0.0193 (19)	0.0062 (14)	−0.0017 (14)	0.0044 (15)
O3	0.036 (4)	0.026 (3)	0.031 (4)	−0.003 (3)	0.005 (3)	0.003 (3)
N3	0.025 (4)	0.023 (3)	0.025 (3)	−0.001 (3)	0.012 (2)	0.007 (2)
C19	0.029 (5)	0.031 (8)	0.035 (8)	0.001 (5)	0.006 (5)	0.017 (7)
C20	0.024 (5)	0.031 (6)	0.025 (5)	0.000 (4)	0.007 (4)	0.007 (4)
C21	0.029 (5)	0.026 (6)	0.026 (6)	0.005 (4)	0.012 (4)	0.003 (4)
O3'	0.036 (4)	0.026 (3)	0.031 (4)	−0.003 (3)	0.005 (3)	0.003 (3)
N3'	0.025 (4)	0.023 (3)	0.025 (3)	−0.001 (3)	0.012 (2)	0.007 (2)
C19'	0.029 (5)	0.031 (8)	0.035 (8)	0.001 (5)	0.006 (5)	0.017 (7)
C20'	0.024 (5)	0.031 (6)	0.025 (5)	0.000 (4)	0.007 (4)	0.007 (4)
C21'	0.029 (5)	0.026 (6)	0.026 (6)	0.005 (4)	0.012 (4)	0.003 (4)

### Geometric parameters (Å, °)

**Table:**

Pb—O2	2.408 (2)	C12—C13	1.403 (6)
Pb—O1	2.468 (2)	C12—H12	0.9500
Pb—N2	2.470 (3)	C13—C14	1.413 (6)
Pb—N1	2.566 (3)	C13—C18	1.421 (5)
Pb—O1^i^	2.618 (2)	C14—C15	1.366 (6)
Pb—O2^ii^	2.812 (2)	C14—H14	0.9500
Pb—O3	2.903 (12)	C15—C16	1.408 (6)
Pb—O3'	2.977 (12)	C15—H15	0.9500
O1—C8	1.317 (4)	C16—C17	1.392 (5)
O1—Pb^i^	2.618 (2)	C16—H16	0.9500
O2—C17	1.304 (4)	C17—C18	1.445 (5)
N1—C1	1.330 (4)	O3—C19	1.244 (10)
N1—C9	1.367 (4)	N3—C19	1.350 (10)
N2—C10	1.319 (5)	N3—C20	1.449 (10)
N2—C18	1.367 (5)	N3—C21	1.448 (10)
C1—C2	1.405 (5)	C19—H19	0.9500
C1—H1	0.9500	C20—H20A	0.9800
C2—C3	1.361 (5)	C20—H20B	0.9800
C2—H2	0.9500	C20—H20C	0.9800
C3—C4	1.412 (5)	C21—H21A	0.9800
C3—H3	0.9500	C21—H21B	0.9800
C4—C5	1.409 (5)	C21—H21C	0.9800
C4—C9	1.429 (5)	O3'—C19'	1.254 (10)
C5—C6	1.361 (6)	N3'—C19'	1.357 (10)
C5—H5	0.9500	N3'—C21'	1.453 (10)
C6—C7	1.410 (5)	N3'—C20'	1.455 (10)
C6—H6	0.9500	C19'—H19'	0.9500
C7—C8	1.387 (5)	C20'—H20D	0.9800
C7—H7	0.9500	C20'—H20E	0.9800
C8—C9	1.439 (5)	C20'—H20F	0.9800
C10—C11	1.402 (7)	C21'—H21D	0.9800
C10—H10	0.9500	C21'—H21E	0.9800
C11—C12	1.366 (8)	C21'—H21F	0.9800
C11—H11	0.9500		
			
O2—Pb—O1	136.81 (8)	C11—C10—H10	118.6
O2—Pb—N2	67.61 (9)	C12—C11—C10	119.0 (4)
O1—Pb—N2	84.94 (9)	C12—C11—H11	120.5
O2—Pb—N1	77.78 (8)	C10—C11—H11	120.5
O1—Pb—N1	65.49 (8)	C11—C12—C13	120.4 (5)
N2—Pb—N1	82.06 (10)	C11—C12—H12	119.8
O2—Pb—O1^i^	133.72 (8)	C13—C12—H12	119.8
O1—Pb—O1^i^	67.90 (9)	C12—C13—C14	123.6 (4)
N2—Pb—O1^i^	80.59 (10)	C12—C13—C18	117.0 (4)
N1—Pb—O1^i^	131.30 (8)	C14—C13—C18	119.4 (4)
C8—O1—Pb	118.6 (2)	C15—C14—C13	119.4 (4)
C8—O1—Pb^i^	129.3 (2)	C15—C14—H14	120.3
Pb—O1—Pb^i^	112.10 (9)	C13—C14—H14	120.3
C17—O2—Pb	119.0 (2)	C14—C15—C16	121.6 (4)
C1—N1—C9	119.1 (3)	C14—C15—H15	119.2
C1—N1—Pb	124.9 (2)	C16—C15—H15	119.2
C9—N1—Pb	114.9 (2)	C17—C16—C15	122.2 (4)
C10—N2—C18	119.0 (4)	C17—C16—H16	118.9
C10—N2—Pb	124.8 (3)	C15—C16—H16	118.9
C18—N2—Pb	116.1 (2)	O2—C17—C16	124.0 (3)
N1—C1—C2	122.8 (3)	O2—C17—C18	119.8 (3)
N1—C1—H1	118.6	C16—C17—C18	116.2 (3)
C2—C1—H1	118.6	N2—C18—C13	121.8 (3)
C3—C2—C1	118.9 (3)	N2—C18—C17	117.0 (3)
C3—C2—H2	120.5	C13—C18—C17	121.3 (3)
C1—C2—H2	120.5	C19—N3—C20	121.2 (19)
C2—C3—C4	120.7 (3)	C19—N3—C21	103.1 (15)
C2—C3—H3	119.6	C20—N3—C21	135.6 (19)
C4—C3—H3	119.6	O3—C19—N3	122.8 (18)
C3—C4—C5	124.4 (3)	O3—C19—H19	118.6
C3—C4—C9	116.8 (3)	N3—C19—H19	118.6
C5—C4—C9	118.8 (3)	C19'—N3'—C21'	118 (2)
C6—C5—C4	120.5 (4)	C19'—N3'—C20'	141 (2)
C6—C5—H5	119.8	C21'—N3'—C20'	101.4 (16)
C4—C5—H5	119.8	O3'—C19'—N3'	123 (2)
C5—C6—C7	120.7 (3)	O3'—C19'—H19'	118.6
C5—C6—H6	119.6	N3'—C19'—H19'	118.6
C7—C6—H6	119.6	N3'—C20'—H20D	109.5
C8—C7—C6	122.4 (3)	N3'—C20'—H20E	109.5
C8—C7—H7	118.8	H20D—C20'—H20E	109.5
C6—C7—H7	118.8	N3'—C20'—H20F	109.5
O1—C8—C7	123.6 (3)	H20D—C20'—H20F	109.5
O1—C8—C9	119.8 (3)	H20E—C20'—H20F	109.5
C7—C8—C9	116.6 (3)	N3'—C21'—H21D	109.5
N1—C9—C4	121.6 (3)	N3'—C21'—H21E	109.5
N1—C9—C8	117.4 (3)	H21D—C21'—H21E	109.5
C4—C9—C8	120.9 (3)	N3'—C21'—H21F	109.5
N2—C10—C11	122.8 (4)	H21D—C21'—H21F	109.5
N2—C10—H10	118.6	H21E—C21'—H21F	109.5
			
O2—Pb—O1—C8	50.9 (3)	C6—C7—C8—C9	0.0 (6)
N2—Pb—O1—C8	100.2 (3)	C1—N1—C9—C4	1.6 (5)
N1—Pb—O1—C8	16.6 (2)	Pb—N1—C9—C4	−167.0 (3)
O1^i^—Pb—O1—C8	−178.0 (3)	C1—N1—C9—C8	−178.3 (3)
O2—Pb—O1—Pb^i^	−131.10 (11)	Pb—N1—C9—C8	13.1 (4)
N2—Pb—O1—Pb^i^	−81.88 (12)	C3—C4—C9—N1	−1.8 (5)
N1—Pb—O1—Pb^i^	−165.45 (14)	C5—C4—C9—N1	177.5 (3)
O1^i^—Pb—O1—Pb^i^	0.0	C3—C4—C9—C8	178.2 (3)
O1—Pb—O2—C17	48.0 (3)	C5—C4—C9—C8	−2.6 (5)
N2—Pb—O2—C17	−6.7 (2)	O1—C8—C9—N1	2.0 (5)
N1—Pb—O2—C17	79.7 (2)	C7—C8—C9—N1	−177.8 (3)
O1^i^—Pb—O2—C17	−57.0 (3)	O1—C8—C9—C4	−177.9 (3)
O2—Pb—N1—C1	20.5 (3)	C7—C8—C9—C4	2.3 (5)
O1—Pb—N1—C1	177.2 (3)	C18—N2—C10—C11	0.3 (8)
N2—Pb—N1—C1	89.2 (3)	Pb—N2—C10—C11	−174.2 (5)
O1^i^—Pb—N1—C1	159.2 (2)	N2—C10—C11—C12	−0.2 (10)
O2—Pb—N1—C9	−171.7 (2)	C10—C11—C12—C13	−0.4 (10)
O1—Pb—N1—C9	−14.9 (2)	C11—C12—C13—C14	−179.3 (5)
N2—Pb—N1—C9	−103.0 (2)	C11—C12—C13—C18	0.7 (8)
O1^i^—Pb—N1—C9	−33.0 (3)	C12—C13—C14—C15	−179.7 (5)
O2—Pb—N2—C10	−179.4 (4)	C18—C13—C14—C15	0.3 (6)
O1—Pb—N2—C10	34.7 (4)	C13—C14—C15—C16	−0.3 (6)
N1—Pb—N2—C10	100.6 (4)	C14—C15—C16—C17	0.3 (6)
O1^i^—Pb—N2—C10	−33.7 (4)	Pb—O2—C17—C16	−174.8 (3)
O2—Pb—N2—C18	6.0 (2)	Pb—O2—C17—C18	6.8 (4)
O1—Pb—N2—C18	−139.9 (3)	C15—C16—C17—O2	−178.8 (3)
N1—Pb—N2—C18	−74.0 (3)	C15—C16—C17—C18	−0.4 (5)
O1^i^—Pb—N2—C18	151.7 (3)	C10—N2—C18—C13	0.0 (6)
C9—N1—C1—C2	−0.5 (5)	Pb—N2—C18—C13	175.1 (3)
Pb—N1—C1—C2	166.9 (3)	C10—N2—C18—C17	179.9 (4)
N1—C1—C2—C3	−0.4 (6)	Pb—N2—C18—C17	−5.1 (4)
C1—C2—C3—C4	0.2 (6)	C12—C13—C18—N2	−0.5 (6)
C2—C3—C4—C5	−178.4 (4)	C14—C13—C18—N2	179.5 (3)
C2—C3—C4—C9	0.8 (5)	C12—C13—C18—C17	179.6 (4)
C3—C4—C5—C6	179.7 (4)	C14—C13—C18—C17	−0.3 (6)
C9—C4—C5—C6	0.5 (5)	O2—C17—C18—N2	−0.9 (5)
C4—C5—C6—C7	1.7 (6)	C16—C17—C18—N2	−179.4 (3)
C5—C6—C7—C8	−2.0 (6)	O2—C17—C18—C13	178.9 (3)
Pb—O1—C8—C7	162.6 (3)	C16—C17—C18—C13	0.4 (5)
Pb^i^—O1—C8—C7	−14.9 (5)	C20—N3—C19—O3	0.0 (3)
Pb—O1—C8—C9	−17.2 (4)	C21—N3—C19—O3	−179.9 (3)
Pb^i^—O1—C8—C9	165.3 (2)	C21'—N3'—C19'—O3'	179.9 (3)
C6—C7—C8—O1	−179.8 (4)	C20'—N3'—C19'—O3'	0.0 (5)

Symmetry codes: (i) −*x*+2, −*y*+1, −*z*+1; (ii) −*x*+1, −*y*+1, −*z*+1.
